# Supplementary material for: Markers of kidney tubule dysfunction and injury and long-term risk of acute kidney injury following coronary artery bypass graft surgery
Source: PLoS One. 2026 Jun 1;21(6):e0331996. doi: 10.1371/journal.pone.0331996 (PMC13225655; doi:10.1371/journal.pone.0331996)
Supplement: S1 Appendix — Models adjusted for age, sex (male and female), race (black and white), urine creatinine, diabetes (yes and no), hypertension (yes and no), BMI, CKD (eGFR < 60 and ≥ 60) and log 2 (urine albumin). Abbreviations: AKI, acute kidney injury; A1M, alpha-1 microglobulin; UMOD, uromodulin; EGF, epidermal growth factor; KIM-1, kidney injury molecule-1; SCr, serum creatinine; eGFR, estimated glomerular filtration rate; OR, odds ratio; CI, confidence interval. (DOCX) [file pone.0331996.s001.docx]

| Biomarker | Time <5 years | Time ≥5 years |
| --- | --- | --- |
| A1M mg/L |  |  |
| Log_2_(A1M) | 1.32 (0.87 – 2.00) | 1.42 (0.92 – 2.20) |
|  |  |  |
| UMOD, pg/mL | |  |
| Log_2_(UMOD) | 0.75 (0.53 – 1.06) | 0.77 (0.59 – 1.01) |
|  |  |  |
| EGF, pg/mL |  |  |
| Log_2_(EGF) | 0.60 (0.38 – 0.94) | 1.00 (0.67 – 1.50) |
|  |  |  |
| KIM-1, pg/mL | |  |
| Log_2_(KIM-1) | 0.66 (0.48 – 0.89) | 1.12 (0.88 – 1.46) |
