# Supplementary material for: Markers of kidney tubule dysfunction and injury and long-term risk of acute kidney injury following coronary artery bypass graft surgery
Source: PLoS One. 2026 Jun 1;21(6):e0331996. doi: 10.1371/journal.pone.0331996 (PMC13225655; doi:10.1371/journal.pone.0331996)
Supplement: Attachment — Submitted filename: Shingler AKI CABG Dysfn Resp Feb 9 2026.docx [file pone.0331996.s002.docx]

PONE-D-25-42789 Kidney Tubule Dysfunction and Injury and the Long-Term Risk of Acute Kidney Injury Following Cardiac Artery Bypass Graft Surgery PLOS ONE

Please submit your revised manuscript by Jan 10 2026 11:59PM. If you will need more time than this to complete your revisions, please reply to this message or contact the journal office at [plosone@plos.org](mailto:plosone@plos.org).

Ramada Rateb Khasawneh
Academic Editor
PLOS ONE

Journal Requirements:

When submitting your revision, we need you to address these additional requirements.

1.Please ensure that your manuscript meets PLOS ONE's style requirements, including those for file naming. The PLOS ONE style templates can be found at

[https://journals.plos.org/plosone/s/file?id=wjVg/PLOSOne_formatting_sample_main_body.pdf](https://urldefense.com/v3/__https:/journals.plos.org/plosone/s/file?id=wjVg*PLOSOne_formatting_sample_main_body.pdf__;Lw!!AU3bcTlGKuA!C0bLaQ28i2WtAmv-3jzByxCfqkUEDF5cawmxR1HJ-lUTMMSQYdIMIQsLewgxRfN0NqWWnNpsRwm9YAgkcTE$) and

[https://journals.plos.org/plosone/s/file?id=ba62/PLOSOne_formatting_sample_title_authors_affiliations.pdf](https://track.editorialmanager.com/CL0/https:%2F%2Fjournals.plos.org%2Fplosone%2Fs%2Ffile%3Fid=ba62%2FPLOSOne_formatting_sample_title_authors_affiliations.pdf/1/010f019abeb6a052-f2b1d442-3aeb-496e-89af-89c92e2ffd58-000000/hv79RUg5eXddSyVJnYRBTApgaGONsQeBeGdaCyVvQGM=239)

- Revised as requested.

2. We note that the grant information you provided in the ‘Funding Information’ and ‘Financial Disclosure’ sections do not match. When you resubmit, please ensure that you provide the correct grant numbers for the awards you received for your study in the ‘Funding Information’ section.

- We updated the funding information. The correct statement is: Supported by award R01-DK128803 from the National Institute of Diabetes and Digestive and Kidney Diseases. The parent REGARDS study was supported by cooperative agreement U01-NS041588 from the National Institute of Neurological Disorders and Stroke (NINDS) and National Institute on Aging (NIA), National Institutes of Health (NIH), Department of Health and Human Services (DHHS). Additional funding was provided by R01-HL80477 and R01-HL165452 from the National Heart Lung and Blood Institute (NHLBI). JVB was funded by R37-DK39773 and R01-DK072381 and U01-DK085660.
- The content is solely the responsibility of the authors and does not necessarily represent the official views of the NINDS or the NIA. Representatives of the NINDS were involved in the review of the manuscript but were not directly involved in the collection, management, analysis or interpretation of the data. The authors thank the other investigators, the staff, and the participants of the REGARDS study for their valuable contributions. A full list of participating REGARDS investigators and institutions can be found at: https://www.uab.edu/soph/regardsstudy/

3. We note that you have indicated that there are restrictions to data sharing for this study. PLOS only allows data to be available upon request if there are legal or ethical restrictions on sharing data publicly. For more information on unacceptable data access restrictions, please see [http://journals.plos.org/plosone/s/data-availability#loc-unacceptable-data-access-restrictions](https://urldefense.com/v3/__http:/journals.plos.org/plosone/s/data-availability*loc-unacceptable-data-access-restrictions__;Iw!!AU3bcTlGKuA!C0bLaQ28i2WtAmv-3jzByxCfqkUEDF5cawmxR1HJ-lUTMMSQYdIMIQsLewgxRfN0NqWWnNpsRwm9hfOyNMU$).

Before we proceed with your manuscript, please address the following prompts:

a) If there are ethical or legal restrictions on sharing a de-identified data set, please explain them in detail (e.g., data contain potentially identifying or sensitive patient information, data are owned by a third-party organization, etc.) and who has imposed them (e.g., a Research Ethics Committee or Institutional Review Board, etc.). Please also provide contact information for a data access committee, ethics committee, or other institutional body to which data requests may be sent.

b) If there are no restrictions, please upload the minimal anonymized data set necessary to replicate your study findings to a stable, public repository and provide us with the relevant URLs, DOIs, or accession numbers. For a list of recommended repositories, please see

[https://journals.plos.org/plosone/s/recommended-repositories](https://urldefense.com/v3/__https:/journals.plos.org/plosone/s/recommended-repositories__;!!AU3bcTlGKuA!C0bLaQ28i2WtAmv-3jzByxCfqkUEDF5cawmxR1HJ-lUTMMSQYdIMIQsLewgxRfN0NqWWnNpsRwm9M1GwS0k$). You also have the option of uploading the data as Supporting Information files, but we would recommend depositing data directly to a data repository if possible.

We will update your Data Availability statement on your behalf to reflect the information you provide.

- The REGARDS study is considered a study in progress, and therefore the data are not publicly available per PLOS practices. Data can be accessed by filing a request with the REGARDS lead center at the University of Alabama at Birmingham and formulating a data use agreement.
- We updated the data access statement using the study’s standard language: **“**REGARDS data are not publicly available due to ethical and legal restrictions. To abide by its obligations with NIH/NINDS and the Institutional Review Board of the University of Alabama at Birmingham, REGARDS facilitates data sharing through data use agreements. Any investigator is welcome to access the REGARDS data, including statistical code, through this process. Requests for data access may be sent to [regardsadmin@uab.edu](mailto:regardsadmin@uab.edu).”
- This terminology was deemed acceptable by your editorial team via email on Dec 10 2025.

4. PLOS requires an ORCID iD for the corresponding author in Editorial Manager on papers submitted after December 6th, 2016. Please ensure that you have an ORCID iD and that it is validated in Editorial Manager. To do this, go to ‘Update my Information’ (in the upper left-hand corner of the main menu), and click on the Fetch/Validate link next to the ORCID field. This will take you to the ORCID site and allow you to create a new iD or authenticate a pre-existing iD in Editorial Manager.

- We added ORCID ID [0000-0002-4738-0093](https://orcid.org/0000-0002-4738-0093) for Dr. Wang, the senior author.

5. Please include your full ethics statement in the ‘Methods’ section of your manuscript file. In your statement, please include the full name of the IRB or ethics committee who approved or waived your study, as well as whether or not you obtained informed written or verbal consent. If consent was waived for your study, please include this information in your statement as well.

- The existing text page 6 line 114 states: “The parent REasons for Geographic and Racial Disparities in Stroke (REGARDS) study and this ancillary study were approved by the Institutional Review Board of the University of Alabama at Birmingham. The parent REGARDS study obtained written consent from all cohort participants, including permission for review of medical records. The IRB waived requirement for informed consent for this ancillary study.”

6. If the reviewer comments include a recommendation to cite specific previously published works, please review and evaluate these publications to determine whether they are relevant and should be cited. There is no requirement to cite these works unless the editor has indicated otherwise.

- Not applicable.

Additional Editor Comments:

Abstract Needs Clearer Structure and Condensation - The abstract is overly detailed, especially the description of the parent cohort and exclusion criteria. This level of procedural detail belongs in the Methods section, not the abstract. The objective and design are clear, but the results could be more concise. Avoid reporting redundant statistics (e.g., demographics already present in the Results section).
The conclusion should more explicitly highlight the clinical implications and novelty (i.e., biomarkers measured years before surgery predict AKI risk).

- We made extensive revisions to the abstract as recommended.

Clarity on Biomarker Categories - There is inconsistency in terminology: A1M and UMOD/EGF are labeled as “tubule dysfunction” markers, while KIM-1 is “injury.” It would help to consistently distinguish these throughout the paper and justify the categorization.
The narrative mixes up “higher quartiles associated with lower odds” and “associated with higher odds”—ensure clarity and correct logical direction.

- We clarified throughout the manuscript that the study pertains to markers of kidney tubule dysfunction and injury.
- We verified the directions of the described associations in the abstract and results. We revised the Results page 14 para 2 to better articulate these associations.

Potential Confounders and Model Justification - Although multivariable models are described, it is unclear whether perioperative characteristics (e.g., bypass time, contrast exposure, pre-op medications) were unavailable or intentionally excluded. Their absence may lead to residual confounding.

- We regret that operative and perioperative information were not available. We clarified this point in the methods and limitations sections. We added comment on page 7 para 2; “Due to limitations in the available records, we were not able to obtain information on perioperative characteristics such as CABG procedure, bypass time, contrast exposure or medications.”

Please justify why the time from REGARDS baseline to CABG (a median of 5.5 years) was treated as a covariate rather than stratified or included as an interaction term, since biomarker stability over time could influence effect estimates.

- **Response**: Accounting for time between measurement of the biomarkers and admission for CABG is important for a range of reasons including drift over time; this is why we included time from baseline to admission in the multivariable-adjusted models.
- In Appendix 1, we provided models stratified by time from REGARDS enrollment to CABG surgery. We modified the Results and added comment in the Discussion (page 20 para 2) to address these results.

Interpretation of KIM-1 Null Findings - KIM-1 is widely considered a sensitive tubular injury marker. The manuscript should more clearly discuss why KIM-1 showed no association in this cohort, especially compared to TRIBE-AKI findings. Possible explanations (biomarker degradation, baseline stability years before surgery, population differences) should be expanded.

- **Response**: It is important to point out that prior studies looking at the association of urine KIM-1 with outcomes (like TRIBE-AKI) largely focused on changes in KIM-1 in the perioperative period as a measure of dynamic tubular injury from the surgical procedure. This is very different from the current study in which urine KIM-1 was measured in community-based individuals at a time of relative health, during which KIM-1’s signal as a sensitive marker of acute tubular injury may have been dampened. This is similar to other studies showing weak or absent associations of KIM-1 with outcomes such as rapid kidney function decline or incident hypertension in ambulatory populations in which it was measured during their healthy baseline. Collectively, we infer from these data that KIM-1 may be most useful as a biomarker of acute tubular injury as opposed to a long-term marker of tubular injury.
- We added a new paragraph with this comment. (Page 19)

Results Section Contains Errors and Ambiguity - There are mismatches in numbers and descriptions. For instance: The statement regarding post-surgery creatinine change appears contradictory (“median 5.5 [4, 8] and 0.1 [0.0, 0.2]”)—this requires correction.
The sentence “higher A1M was associated with lower odds of AKI” contradicts the OR 1.34 (which indicates higher odds). These inconsistencies reduce confidence in the reported findings.

- We apologize for the oversights. We have made appropriate edits throughout the results section.

Missing Discussion on Clinical Utility - The paper asserts that biomarkers may allow “early identification,” but the effect sizes are modest and CIs include the null (e.g., A1M lower bound 1.00). The discussion should temper conclusions and describe whether these biomarkers provide predictive value beyond existing models.

- We clarified on Page 20 para 1; “We underscore the preliminary nature of the current study. Independent validation with a larger cohort is necessary to verify these results.”

Strengths/Limitations Need Stronger Integration with Findings

- We revised as suggested. Please see page 21.

The manuscript mentions biomarker degradation, restricted age range, and lack of pre-hospitalization creatinine—but does not explain how these factors may bias results.
Consider including a paragraph addressing the generalizability to younger populations and whether exclusion of 48% of CABG records (due to illegibility/incompleteness) may introduce selection bias.

- We provided additional comments on these points in the limitations section. Please see Page 21 para 1

Use consistent terms for CABG (either “CABG surgery” or “CABG”) throughout.--> Standardize “peri-CABG AKI,” “post-CABG AKI,” and “AKI following CABG.”

- We aligned all terminology through the paper as suggested.

[Note: HTML markup is below. Please do not edit.]

Reviewers' comments:

Reviewer's Responses to Questions

**Comments to the Author**

1. Is the manuscript technically sound, and do the data support the conclusions?

The manuscript must describe a technically sound piece of scientific research with data that supports the conclusions. Experiments must have been conducted rigorously, with appropriate controls, replication, and sample sizes. The conclusions must be drawn appropriately based on the data presented.

Reviewer #1: Yes

Reviewer #2: Yes

2. Has the statistical analysis been performed appropriately and rigorously?

Reviewer #1: Yes

Reviewer #2: I Don't Know

3. Have the authors made all data underlying the findings in their manuscript fully available?

The [PLOS Data policy](http://track.editorialmanager.com/CL0/http:%2F%2Fwww.plosone.org%2Fstatic%2Fpolicies.action%23sharing/1/010f019abeb6a052-f2b1d442-3aeb-496e-89af-89c92e2ffd58-000000/Li5mT9EZ9xN2rf9iZqVWzRAHshfn72s1aoRmcgk8wvU=239) requires authors to make all data underlying the findings described in their manuscript fully available without restriction, with rare exception (please refer to the Data Availability Statement in the manuscript PDF file). The data should be provided as part of the manuscript or its supporting information, or deposited to a public repository. For example, in addition to summary statistics, the data points behind means, medians and variance measures should be available. If there are restrictions on publicly sharing data—e.g. participant privacy or use of data from a third party—those must be specified.

Reviewer #1: Yes

Reviewer #2: No

- Please see response regarding updated data access statement.

4. Is the manuscript presented in an intelligible fashion and written in standard English?

PLOS ONE does not copyedit accepted manuscripts, so the language in submitted articles must be clear, correct, and unambiguous. Any typographical or grammatical errors should be corrected at revision, so please note any specific errors here.

Reviewer #1: Yes

Reviewer #2: Yes

5. Review Comments to the Author

Please use the space provided to explain your answers to the questions above. You may also include additional comments for the author, including concerns about dual publication, research ethics, or publication ethics. (Please upload your review as an attachment if it exceeds 20,000 characters)

Reviewer #1: I recommend the acceptance of this manuscript for publication. The study presents a novel and significant contribution to the field of nephrology and cardiac surgery by investigating the long-term predictive value of kidney tubular biomarkers for acute kidney injury (AKI) following coronary artery bypass graft (CABG) surgery. Its prospective design, rigorous methodology, and insightful findings offer a new paradigm for understanding AKI susceptibility, moving beyond immediate perioperative risk factors to assess renal health years in advance. The work is well-executed, clearly written, and has substantial potential to influence future clinical practice and research directions.

This study's core contribution lies in its innovative temporal approach, evaluating kidney tubular health at a stable baseline an average of 5.5 years before the stressor event, thus framing CABG as a "renal stress test" to uncover subclinical vulnerability. The research design is robust, nested within the large, population-based REGARDS cohort, and employs a well-defined cohort of 394 patients who underwent CABG. The methodology is commendable, utilizing precise measurements of specific biomarkers for tubular dysfunction (A1M, UMOD, EGF) and injury (KIM-1) and applying rigorous statistical models that adjust for a comprehensive set of confounders, including traditional markers like eGFR and albuminuria. The key findings are compelling: higher baseline A1M was associated with increased odds of AKI, while higher UMOD was associated with decreased odds, suggesting that markers of tubular dysfunction, rather than injury, are potent long-term predictors. Clinically, this opens a vital window for early risk stratification, paving the way for targeted preventive strategies to mitigate AKI risk in vulnerable patients undergoing cardiac surgery.

To further strengthen this already impactful manuscript, I offer the following specific suggestions for revision:

1.Enhance Clinical Context: In the discussion, please elaborate on the practical feasibility and potential cost-effectiveness of implementing these biomarker tests in routine pre-operative assessment pathways. A brief comparison of their predictive power against existing clinical risk models could also be valuable for clinicians.

- **Response**: Although beyond the scope of the current manuscript, a potentially exciting implication of these findings is the future ability to use urine biomarkers to help risk stratify patients undergoing surgical or other procedures that may lead to kidney physiologic stress. Using these biomarkers to help quantify the potential risk of AKI in individuals prior to the procedure (presumably during a period of relative health) could aid in shared decision making about the risks of the procedure and any pre-emptive measures that might be taken beforehand, such as early admission for optimization of kidney health. We have added this clinical context to the revised manuscript.
- We strengthened comment in the discussion section. Please see page 20 para 2.

2.Address Surgical Heterogeneity: While the study acknowledges limitations regarding surgical details, a brief discussion in the limitations section on how the lack of differentiation between elective and urgent cases, or variations in bypass time, might influence the observed associations would add nuance.

- Regrettably, information on surgical heterogeneity was not available. We added comment in the Limitations section.

3.Clarify Biomarker Stability: The potential for biomarker degradation over long-term storage is mentioned as a limitation. It would be helpful to briefly cite any existing literature on the long-term stability of these specific urinary biomarkers under the storage conditions described, to better reassure the reader about the validity of the measurements.

- We added comment and references to Limitations Page 21 para 1 that degradation of the markers could have biased the results to null.

4.Future Directions - Specificity: The suggestion for future research is excellent. To make it more concrete, consider proposing a specific follow-up study, such as a prospective trial where high-risk patients identified by these biomarkers are randomized to a KDIGO-guided preventive bundle versus standard care to assess outcomes.

- We expanded discussion to include a concept for a future study. Please see revised page 20, para 2.

5.Additional discussion: The author can further explore in the discussion section the detectability, cost-effectiveness of markers such as A1M in clinical practice, as well as their potential for integration with existing clinical risk prediction models (such as the Demirjian model).

- See answer above

Reviewer #2: The manuscript is an adequate scientific research. It is written in standard English. Requirement of informed consent has been waived. however, regarding data availability, some restrictions has been applied

6. PLOS authors have the option to publish the peer review history of their article ([what does this mean?](https://track.editorialmanager.com/CL0/https:%2F%2Fjournals.plos.org%2Fplosone%2Fs%2Feditorial-and-peer-review-process%23loc-peer-review-history/1/010f019abeb6a052-f2b1d442-3aeb-496e-89af-89c92e2ffd58-000000/0kJfrFiBz9x7jQK-wR0F1bs9DjmkEO7L3XSJtfnOl5k=239)). If published, this will include your full peer review and any attached files.

If you choose “no”, your identity will remain anonymous but your review may still be made public.


**Do you want your identity to be public for this peer review?** For information about this choice, including consent withdrawal, please see our [Privacy Policy](https://track.editorialmanager.com/CL0/https:%2F%2Fwww.plos.org%2Fprivacy-policy/1/010f019abeb6a052-f2b1d442-3aeb-496e-89af-89c92e2ffd58-000000/cZnD9guUGVpwi_kjfGnJpVY1SJe7MDeYbEqxup8JjP0=239).

Reviewer #1: No

Reviewer #2: No

 [NOTE: If reviewer comments were submitted as an attachment file, they will be attached to this email and accessible via the submission site. Please log into your account, locate the manuscript record, and check for the action link "View Attachments". If this link does not appear, there are no attachment files.]

To ensure your figures meet our technical requirements, please review our figure guidelines: [https://journals.plos.org/plosone/s/figures](https://urldefense.com/v3/__https:/journals.plos.org/plosone/s/figures__;!!AU3bcTlGKuA!C0bLaQ28i2WtAmv-3jzByxCfqkUEDF5cawmxR1HJ-lUTMMSQYdIMIQsLewgxRfN0NqWWnNpsRwm9Sx3x_KY$)

You may also use PLOS’s free figure tool, NAAS, to help you prepare publication quality figures: [https://journals.plos.org/plosone/s/figures#loc-tools-for-figure-preparation](https://urldefense.com/v3/__https:/journals.plos.org/plosone/s/figures*loc-tools-for-figure-preparation__;Iw!!AU3bcTlGKuA!C0bLaQ28i2WtAmv-3jzByxCfqkUEDF5cawmxR1HJ-lUTMMSQYdIMIQsLewgxRfN0NqWWnNpsRwm92GZAtCc$).

NAAS will assess whether your figures meet our technical requirements by comparing each figure against our figure specifications.

*In compliance with data protection regulations, you may request that we remove your personal registration details at any time.* [*(Remove my information/details)*](https://track.editorialmanager.com/CL0/https:%2F%2Fwww.editorialmanager.com%2Fpone%2Flogin.asp%3Fa=r/1/010f019abeb6a052-f2b1d442-3aeb-496e-89af-89c92e2ffd58-000000/jt5I0mZ0NSDcfJh9LP5wFlXSgQ6500gPAQ_LwB7JDZA=239)*. Please contact the publication office if you have any questions.*
