# Supplementary material for: Markers of kidney tubule dysfunction and injury and long-term risk of acute kidney injury following coronary artery bypass graft surgery
Source: PLoS One. 2026 Jun 1;21(6):e0331996. doi: 10.1371/journal.pone.0331996 (PMC13225655; doi:10.1371/journal.pone.0331996)
Supplement: Attachment — Submitted filename: Reviewer matrixBiomarkersKidney.docx [file pone.0331996.s003.docx]

**REVIEWER’S COMMENTS**

PLOS Global Public Health

Markers of kidney tubule dysfunction and injury and long-term risk of acute kidney injury following cardiac artery bypass graft surgery

| **S/N** | **Section** | **Comments from the reviewer** | **PI’s response** | **Line number** |
| --- | --- | --- | --- | --- |
| 1. | **Title** | OK | NA | NA |
| 2. | **Abstract** | Your abstract section has no an introductory part |  | 47-76 |
|  |  | You have a lot of sub-section in your abstract please arrange them in a good format |  | 47-76 |
| 3. | **Introduction** | Please incooporate in your introduction the magnitude globally, region wise and locally |  | 78-108 |
|  |  | Please give more detailed background of your introduction |  | 78-108 |
| 4. | **Materials and Methods** | Please there are overlapping passages under this section please have a look into it. For example, You can not have design section at the same time speaking ethical clearance and consent in one section |  | 111-135 |
|  |  | Please provide long form for the word “Lab” |  | 185 |
|  |  | Does your analysis included “Confidence Intervals” if yes please incoorporate into the analysis section |  | 191-205 |
| 5. | **Results** | Please explain only the result under this section do not mix explanation into one section |  | 207-216 |
| 6. | **Discussion** | Discus your results with supporting evidence from other studies. |  | 310-390 |
|  |  | Meanwhile discuss your results by connecting to your objectives |  | 310-390 |
| 7 | **References** | You have used very outdated references. Please re-check them |  | 410-543 |
| 8. | **General comments and Questions** | Please have separate section for strengths and limitations |  |  |
|  |  | How was confidentiality of the study be maintained please incoorporate this statement |  |  |
|  |  | Your manuscript has overlapping passages in the introduction and methods sections. Please rephrase them |  |  |
|  |  | Was it necessary to include recommendations in this study? |  |  |
|  |  | Please add each authors contribution in your manuscript. |  |  |
|  |  | Consider proper numbering in your manuscript |  |  |
|  |  | Please revise how to write manuscript properly as many of the important parts were missing? For example in the authors details you provided an email contact which is not proper |  |  |
