# Supplementary material for: Markers of kidney tubule dysfunction and injury and long-term risk of acute kidney injury following coronary artery bypass graft surgery
Source: PLoS One. 2026 Jun 1;21(6):e0331996. doi: 10.1371/journal.pone.0331996 (PMC13225655; doi:10.1371/journal.pone.0331996)
Supplement: Attachment — Submitted filename: AKI CABG Dys Responses Apr 9 2026.docx [file pone.0331996.s004.docx]

Reponses to Comments

Reviewer #3: The authors have tried to respond to many questions. But some of the sections have overlapped to each other.

- We apologize – the nature of this comment is not clear. We believe that the relative sections of the paper are distinct and have been appropriately separated.

Reviewer #4: Thank you for the revision. The manuscript is improved and most substantive concerns have been addressed. However, the clean revised version still contains several obvious internal errors that should be corrected before acceptance. The main remaining issues are: (1) the title uses “cardiac artery bypass graft” instead of “coronary artery bypass graft”;

- We apologize for this oversight. We have made appropriate edits.

(2) the cohort size is given as 30,239 in the manuscript background but 30,329 in the Results;

- The correct number is 30,239. We have made appropriate edits.

(3) the Figure 1 legend refers to the “REGARDS Trial” rather than the REGARDS cohort/study;

- We apologize. We have corrected the caption to “REGARDS cohort.”

(4) Table 3 appears to contain an impossible EGF summary value for the AKI group (6.6 [4.0–1.1]);

- We corrected the errant value. We took this opportunity to verify all numbers. There are a few small corrections in the tables and appendix.

(5) at least one Results sentence remains garbled (“lower higher odds,” “while whereas”); and

- We corrected page 14 para 1.

(6) the PrevAKI trial is described as ongoing even though the cited study is a published randomized trial. These look fixable and do not seem to undermine the overall scientific message, but they reflect insufficient final quality control. I would therefore recommend revision rather than acceptance in the current form.

- We apologize for the oversight. Please see corrected page 19 para 2. We revised the relevant sentence to “Among cardiac surgery patients identified as high-risk patients using urinary biomarkers [TIMP-2]*[IGFBP7], the PrevAKI trial demonstrated that applying a KDIGO care bundle reduced the incidence and severity of post-operative AKI compared with standard care.”

| **S/N** | **Section** | **Comments from the reviewer** | **PI’s response** | **Line number** |
| --- | --- | --- | --- | --- |
| 1. | **Title** | OK | NA | NA |
| 2. | **Abstract** | Your abstract section has no an introductory part | We revised the abstract to contain an introductory statement. “Approximately one in five patients undergoing coronary artery bypass graft (CABG) surgery will develop post-operative acute kidney injury (AKI).” | 47-76 |
|  |  | You have a lot of sub-section in your abstract please arrange them in a good format | We revised the abstract to adhere to PLOS One examples | 47-76 |
| 3. | **Introduction** | Please incooporate in your introduction the magnitude globally, region wise and locally | We revised as suggested. Please see manuscript. | 78-108 |
|  |  | Please give more detailed background of your introduction | We revised as suggested. Please see manuscript. | 78-108 |
| 4. | **Materials and Methods** | Please there are overlapping passages under this section please have a look into it. For example, You can not have design section at the same time speaking ethical clearance and consent in one section | We appreciate the comment. We believe that as written, the methods section adequately separates the various parts of the scientific approach, without overlap.  In deference to the comment, we broke out “Ethics Approval and Informed Consent” into a separate subsection. | 111-135 |
|  |  | Please provide long form for the word “Lab” | We corrected the spelling. | 185 |
|  |  | Does your analysis included “Confidence Intervals” if yes please incoorporate into the analysis section | In the last paragraph of the methods section, we added “Statistical significance was set at 0.05 (two-tailed), and we calculated 95% confidence intervals for all odds ratios.” | 191-205 |
| 5. | **Results** | Please explain only the result under this section do not mix explanation into one section | We appreciate the suggestion. We verified that the results section is not interpretive or explanatory. | 207-216 |
| 6. | **Discussion** | Discus your results with supporting evidence from other studies. | We significantly expanded page 21, para 2, with additional relevant discussion. | 310-390 |
|  |  | Meanwhile discuss your results by connecting to your objectives | We believe that the discussion adequately connects with the stated objectives. We believe that the existing text has strong linkages to existing evidence. | 310-390 |
| 7 | **References** | You have used very outdated references. Please re-check them | While our reference list includes several foundational studies, the manuscript is primarily supported by recent literature (2021–2024), and older citations were retained only where they represent seminal work that remains central to current understanding and continues to be cited in contemporary reviews and guidelines. | 410-543 |
| 8. | **General comments and Questions** | Please have separate section for strengths and limitations | Our quick review of other PLOS ONE papers suggests that “strengths” and “limitations” are not standard journal subsections. Therefore, we opted to omit these subheadings. |  |
|  |  | How was confidentiality of the study be maintained please incoorporate this statement | We added to the methods page 7 para 1; “The identities of the study participants were confidential and not accessible to the investigators.” |  |
|  |  | Your manuscript has overlapping passages in the introduction and methods sections. Please rephrase them | We believe that as written, the methods section adequately separates the various parts of the scientific approach, without overlap |  |
|  |  | Was it necessary to include recommendations in this study? | We believe that the current Discussion text is appropriate and not over-reaching. |  |
|  |  | Please add each authors contribution in your manuscript. | We added author contributions to the title page. |  |
|  |  | Consider proper numbering in your manuscript | It is not clear what this comment refers to. All sections appear to be correctly numbered. |  |
|  |  | Please revise how to write manuscript properly as many of the important parts were missing? For example in the authors details you provided an email contact which is not proper | The title page and email contact are formatted according to PLOS One standards. |  |
